# *catena*-Poly[[tripyridine­nickel(II)]-μ-5-bromo­isophthalato]

**DOI:** 10.1107/S1600536808018916

**Published:** 2008-06-28

**Authors:** Lijun Liu

**Affiliations:** a Department of Chemistry, Liaocheng University, Liaocheng, Shandong 252059, People’s Republic of China

## Abstract

The title compound, [Ni(C_8_H_3_BrO_4_)(C_5_H_5_N)_3_], is the first structurally characterized complex with a transition metal coordinated by a 5-bromo­isophthalate anion. The Ni^II^ ion is coordinated by three O atoms from the carboxyl­ate groups and three N atoms from three pyridine ligands in a distorted octa­hedral coordination geometry. The 5-bromo­isophthalate anion is planar within 0.057 (2) Å. The two carboxyl­ate groups of the ligand coordinate the Ni^II^ ions in a chelating and monodentate mode, linking the metal atoms into infinite chains along the [010] direction. These chains are stacked together *via* strong π–π inter­actions between the pyridine rings [centroid–centroid distance 3.601 (4) Å], forming a three-dimensional motif.

## Related literature

For related literature, see: Therrien *et al.* (2005 ▶). 
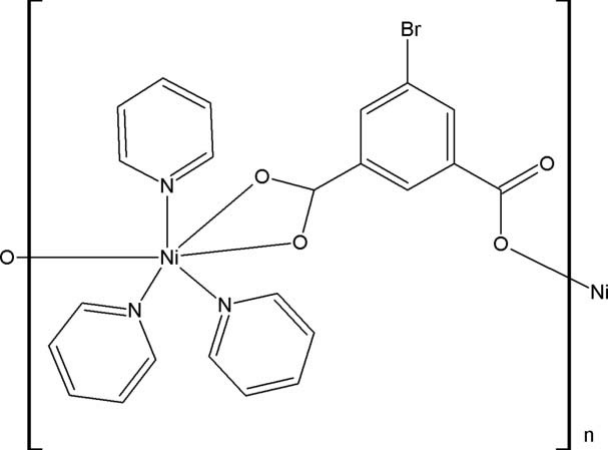


## Experimental

### 

#### Crystal data

[Ni(C_8_H_3_BrO_4_)(C_5_H_5_N)_3_]
*M* *_r_* = 539.02
Monoclinic, 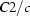

*a* = 19.6621 (6) Å
*b* = 16.0190 (6) Å
*c* = 14.8755 (5) Å
β = 111.504 (2)°
*V* = 4359.2 (3) Å^3^
*Z* = 8
Mo *K*α radiation
μ = 2.76 mm^−1^
*T* = 296 (2) K
0.22 × 0.20 × 0.08 mm

#### Data collection

Bruker SMART 1K CCD area-detector diffractometer
Absorption correction: multi-scan (*SADABS*; Sheldrick, 1996 ▶) *T*_min_ = 0.582, *T*_max_ = 0.809
22727 measured reflections
5402 independent reflections
4195 reflections with *I* > 2σ(*I*)
*R*_int_ = 0.034

#### Refinement

*R*[*F* ^2^ > 2σ(*F* ^2^)] = 0.036
*wR*(*F* ^2^) = 0.096
*S* = 1.05
5402 reflections
289 parameters
H-atom parameters constrained
Δρ_max_ = 0.65 e Å^−3^
Δρ_min_ = −0.34 e Å^−3^

### 

Data collection: *SMART* (Bruker, 2007 ▶); cell refinement: *SAINT-Plus* (Bruker, 2007 ▶); data reduction: *SAINT-Plus*; program(s) used to solve structure: *SHELXS97* (Sheldrick, 2008 ▶); program(s) used to refine structure: *SHELXL97* (Sheldrick, 2008 ▶); molecular graphics: *SHELXTL* (Sheldrick, 2008 ▶); software used to prepare material for publication: *SHELXTL*.

## Supplementary Material

Crystal structure: contains datablocks global, I. DOI: 10.1107/S1600536808018916/fj2127sup1.cif

Structure factors: contains datablocks I. DOI: 10.1107/S1600536808018916/fj2127Isup2.hkl

Additional supplementary materials:  crystallographic information; 3D view; checkCIF report

## Figures and Tables

**Table:** 

Br1—C5	1.899 (2)
Ni1—N2	2.1439 (19)
Ni1—O2	2.1655 (17)
Ni1—O1	2.2024 (17)

**Table:** 

N2—Ni1—O2	152.98 (7)
N2—Ni1—O1	92.78 (7)
O2—Ni1—O1	60.20 (6)
N2—Ni1—N1	90.11 (7)
O2—Ni1—N1	90.48 (7)
O1—Ni1—N1	90.02 (7)
N2—Ni1—N3	91.78 (7)
O2—Ni1—N3	88.13 (7)
O1—Ni1—N3	90.54 (7)
N1—Ni1—N3	178.00 (7)

**Fig. 1.**
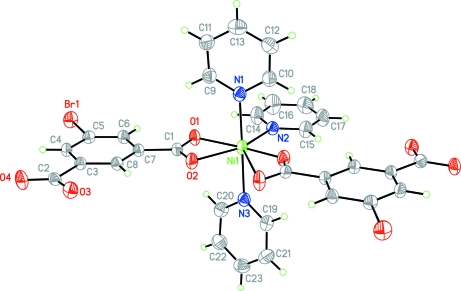
Molecular structure showing 50% probability displacement ellipsoids. The unlabeled atoms are derived from the reference atoms by means of the (1.5 - x, -1/2 + y, 1.5 - z) symmetry transformation..

**Fig. 2.**
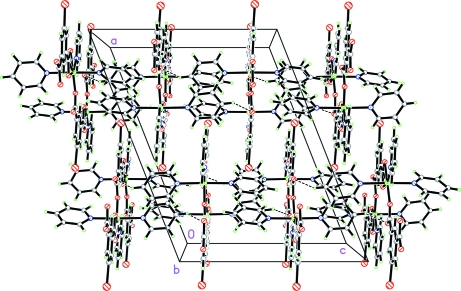
Packing diagram viewed down the b axis,

## supplementary crystallographic information

### Comment

The 5-bromoisophthalic acid ligand (H_2_BIPA) was hitherto reported in its molecular form in the cystal structure of its cocrystal complex (Therrien *et al.*, 2005). This paper provides the fist example of its structurally characterized complex with a transition metal; the ligand in this complex is twice deprotonated.

The asymmetric unit of Ni(BIPA)(C_5_H_5_N)_3_ occupies a general position in the unit cell; the Ni atom is coordinated by four O atoms from the carboxylicate groups and three N atoms from the pyridine ligands (Ni1—N2 2.143 (2), Ni1—O2 2.165 (2), Ni1—O1 2.202 (2), Ni1—N1 2.204 (2), Ni1—N3 2.204 (2) and Ni1—O3 1.996 (2) Å) (Fig. 1). The BIPA ligand has essentially planar conformation, the maximum deviation of the O3 atom from its mean plane being 0.057 (2) Å. The geometry of the ligand is similar to the one observed in Therrien *et al.*, (2005).

No guest molecule or Hydrogen bond was detected in the structure; The two carboxylate groups of the ligand coordinated with the Ni(II) in monodentate and chelated mode, respectively, linking the Ni(II) ion into an infinte chain along the (010) direction. The chains are stacked together *via* the strong π-π interactions between the pyridine rings to form a three-dimensional motif.(Fig. 2).

### Experimental

NiCl_2_.4H_2_O (0.5 mmol, 101 mg) and 5-bromoisophthalic acid (0.5 mmol, 123 mg) were added to 30 ml of distilled water. After stirring for 15 min at room temperature, the pH value was adjusted to 6 by few drops of pyridine, and clear solution was allowed to evaporate in the ventilating cabinet. Light green plate crystals of the title compound were obtained after 4 days, in yields of 35%. The crystals were filtered, washed by cold EtOH and dried in the air.

### Refinement

All of the H atoms were positioned geometrically and refined using a riding model with C—H = 0.930 Å, with *U*_iso_(H) = 1.2 times *U*_eq_(C).

### Crystal data

**Table:**

[Ni(C_8_H_3_BrO_4_)(C_5_H_5_N)_3_]	*F*_000_ = 2176
*M**_r_* = 539.02	*D*_x_ = 1.643 Mg m^−^^3^
Monoclinic, *C*2/*c*	Mo *K*α radiation λ = 0.71073 Å
Hall symbol: -C 2yc	Cell parameters from 9035 reflections
*a* = 19.6621 (6) Å	θ = 2.7–28.7º
*b* = 16.0190 (6) Å	µ = 2.76 mm^−^^1^
*c* = 14.8755 (5) Å	*T* = 296 (2) K
β = 111.504 (2)º	Plate, green
*V* = 4359.2 (3) Å^3^	0.22 × 0.20 × 0.08 mm
*Z* = 8	

### Data collection

**Table:**

Bruker SMART 1K CCD area-detector diffractometer	5402 independent reflections
Radiation source: fine-focus sealed tube	4195 reflections with *I* > 2σ(*I*)
Monochromator: graphite	*R*_int_ = 0.034
*T* = 296(2) K	θ_max_ = 28.3º
φ and ω scans	θ_min_ = 2.0º
Absorption correction: multi-scan(SADABS; Sheldrick, 1996)	*h* = −26→26
*T*_min_ = 0.582, *T*_max_ = 0.809	*k* = −21→21
22727 measured reflections	*l* = −19→19

### Refinement

**Table:**

Refinement on *F*^2^	Secondary atom site location: difference Fourier map
Least-squares matrix: full	Hydrogen site location: inferred from neighbouring sites
*R*[*F*^2^ > 2σ(*F*^2^)] = 0.036	H-atom parameters constrained
*wR*(*F*^2^) = 0.096	*w* = 1/[σ^2^(*F*_o_^2^) + (0.0529*P*)^2^ + 2.0236*P*] where *P* = (*F*_o_^2^ + 2*F*_c_^2^)/3
*S* = 1.05	(Δ/σ)_max_ < 0.001
5402 reflections	Δρ_max_ = 0.65 e Å^−^^3^
289 parameters	Δρ_min_ = −0.34 e Å^−^^3^
Primary atom site location: structure-invariant direct methods	

### Special details

**Table:**

Geometry. All e.s.d.'s (except the e.s.d. in the dihedral angle between two l.s. planes) are estimated using the full covariance matrix. The cell e.s.d.'s are taken into account individually in the estimation of e.s.d.'s in distances, angles and torsion angles; correlations between e.s.d.'s in cell parameters are only used when they are defined by crystal symmetry. An approximate (isotropic) treatment of cell e.s.d.'s is used for estimating e.s.d.'s involving l.s. planes.
Refinement. Refinement of *F*^2^ against ALL reflections. The weighted *R*-factor *wR* and goodness of fit *S* are based on *F*^2^, conventional *R*-factors *R* are based on *F*, with *F* set to zero for negative *F*^2^. The threshold expression of *F*^2^ > σ(*F*^2^) is used only for calculating *R*-factors(gt) *etc*. and is not relevant to the choice of reflections for refinement. *R*-factors based on *F*^2^ are statistically about twice as large as those based on *F*, and *R*- factors based on ALL data will be even larger.

### Fractional atomic coordinates and isotropic or equivalent isotropic displacement parameters (Å^2^)

**Table:**

	*x*	*y*	*z*	*U*_iso_*/*U*_eq_	
Br1	1.104271 (13)	1.027190 (17)	0.93289 (2)	0.04600 (10)	
Ni1	0.833627 (16)	0.667412 (18)	0.78863 (2)	0.03305 (10)	
O1	0.92550 (9)	0.75627 (10)	0.83248 (13)	0.0385 (4)	
N1	0.83251 (11)	0.66980 (12)	0.93619 (15)	0.0352 (4)	
C1	0.87970 (13)	0.81533 (14)	0.80832 (16)	0.0313 (5)	
O2	0.81214 (9)	0.80030 (10)	0.77426 (13)	0.0386 (4)	
N2	0.90493 (11)	0.56123 (12)	0.82571 (14)	0.0333 (4)	
C2	0.82483 (13)	1.12204 (14)	0.78305 (16)	0.0314 (5)	
O3	0.75769 (9)	1.09924 (11)	0.75262 (12)	0.0396 (4)	
N3	0.83124 (11)	0.66766 (12)	0.63935 (15)	0.0351 (4)	
C3	0.87912 (12)	1.05099 (14)	0.81091 (16)	0.0282 (4)	
O4	0.84705 (11)	1.19426 (11)	0.79268 (15)	0.0487 (5)	
C4	0.95349 (12)	1.06846 (14)	0.85107 (16)	0.0309 (5)	
H4	0.9698	1.1234	0.8609	0.037*	
C5	1.00279 (12)	1.00317 (15)	0.87614 (16)	0.0311 (5)	
C6	0.98030 (12)	0.92097 (14)	0.86223 (17)	0.0318 (5)	
H6	1.0143	0.8778	0.8796	0.038*	
C7	0.90594 (12)	0.90378 (13)	0.82181 (16)	0.0291 (5)	
C8	0.85606 (13)	0.96895 (14)	0.79651 (16)	0.0298 (5)	
H8	0.8063	0.9572	0.7694	0.036*	
C9	0.85644 (15)	0.73386 (16)	0.99719 (19)	0.0425 (6)	
H9	0.8809	0.7770	0.9799	0.051*	
C10	0.79950 (15)	0.60768 (16)	0.9655 (2)	0.0426 (6)	
H10	0.7834	0.5614	0.9256	0.051*	
C11	0.84689 (16)	0.73964 (17)	1.0846 (2)	0.0467 (6)	
H11	0.8646	0.7855	1.1247	0.056*	
C12	0.78831 (15)	0.60906 (19)	1.0510 (2)	0.0481 (7)	
H12	0.7655	0.5644	1.0684	0.058*	
C13	0.81117 (16)	0.67711 (18)	1.1112 (2)	0.0479 (7)	
H13	0.8025	0.6803	1.1685	0.058*	
C14	0.97593 (15)	0.57400 (17)	0.8668 (2)	0.0509 (7)	
H14	0.9924	0.6288	0.8794	0.061*	
C15	0.88262 (15)	0.48241 (16)	0.8088 (2)	0.0452 (6)	
H15	0.8328	0.4721	0.7791	0.054*	
C16	1.02719 (16)	0.5108 (2)	0.8921 (3)	0.0647 (9)	
H16	1.0768	0.5228	0.9200	0.078*	
C17	0.92961 (17)	0.41524 (17)	0.8331 (2)	0.0520 (7)	
H17	0.9118	0.3609	0.8213	0.062*	
C18	1.00334 (17)	0.43030 (19)	0.8752 (2)	0.0569 (8)	
H18	1.0365	0.3863	0.8918	0.068*	
C19	0.77634 (15)	0.63156 (16)	0.56745 (19)	0.0419 (6)	
H19	0.7427	0.6000	0.5835	0.050*	
C20	0.87919 (15)	0.71115 (17)	0.6137 (2)	0.0434 (6)	
H20	0.9185	0.7359	0.6621	0.052*	
C21	0.76665 (16)	0.63834 (18)	0.4714 (2)	0.0478 (7)	
H21	0.7276	0.6118	0.4244	0.057*	
C22	0.87340 (17)	0.72126 (19)	0.5186 (2)	0.0504 (7)	
H22	0.9080	0.7522	0.5040	0.061*	
C23	0.81539 (16)	0.68473 (18)	0.4458 (2)	0.0488 (7)	
H23	0.8096	0.6914	0.3813	0.059*	

### Atomic displacement parameters (Å^2^)

**Table:**

	*U*^11^	*U*^22^	*U*^33^	*U*^12^	*U*^13^	*U*^23^
Br1	0.02529 (13)	0.04761 (17)	0.05865 (18)	−0.00510 (11)	0.00777 (11)	−0.00334 (12)
Ni1	0.02778 (16)	0.02420 (15)	0.04311 (19)	−0.00138 (11)	0.00821 (13)	−0.00097 (12)
O1	0.0380 (9)	0.0225 (8)	0.0521 (10)	0.0005 (7)	0.0130 (8)	−0.0004 (7)
N1	0.0341 (11)	0.0288 (10)	0.0404 (11)	0.0037 (8)	0.0107 (9)	−0.0003 (8)
C1	0.0334 (12)	0.0258 (11)	0.0337 (12)	−0.0037 (9)	0.0113 (10)	0.0000 (9)
O2	0.0331 (9)	0.0283 (8)	0.0503 (10)	−0.0051 (7)	0.0103 (8)	−0.0015 (7)
N2	0.0304 (10)	0.0277 (9)	0.0387 (11)	0.0032 (8)	0.0091 (8)	0.0007 (8)
C2	0.0350 (12)	0.0280 (11)	0.0306 (11)	0.0075 (9)	0.0114 (10)	0.0030 (9)
O3	0.0276 (9)	0.0359 (9)	0.0488 (10)	0.0089 (7)	0.0063 (7)	0.0004 (8)
N3	0.0340 (11)	0.0285 (10)	0.0404 (11)	0.0035 (8)	0.0109 (9)	0.0002 (8)
C3	0.0281 (11)	0.0254 (10)	0.0312 (11)	0.0016 (8)	0.0108 (9)	0.0001 (9)
O4	0.0485 (11)	0.0266 (9)	0.0697 (13)	0.0064 (8)	0.0203 (10)	0.0038 (8)
C4	0.0316 (12)	0.0230 (10)	0.0371 (12)	−0.0023 (9)	0.0116 (10)	−0.0016 (9)
C5	0.0215 (10)	0.0338 (11)	0.0343 (12)	−0.0012 (9)	0.0061 (9)	−0.0003 (9)
C6	0.0267 (11)	0.0272 (11)	0.0384 (12)	0.0050 (9)	0.0084 (9)	0.0016 (9)
C7	0.0300 (11)	0.0242 (10)	0.0324 (11)	0.0006 (9)	0.0107 (9)	0.0006 (9)
C8	0.0257 (11)	0.0272 (11)	0.0350 (12)	−0.0004 (9)	0.0094 (9)	0.0011 (9)
C9	0.0464 (15)	0.0333 (13)	0.0454 (15)	0.0002 (11)	0.0140 (12)	−0.0009 (11)
C10	0.0405 (14)	0.0361 (13)	0.0466 (15)	−0.0023 (11)	0.0105 (11)	0.0017 (11)
C11	0.0585 (17)	0.0363 (14)	0.0412 (14)	0.0086 (13)	0.0136 (13)	−0.0008 (11)
C12	0.0406 (15)	0.0534 (16)	0.0490 (16)	0.0000 (13)	0.0149 (12)	0.0109 (13)
C13	0.0452 (15)	0.0554 (17)	0.0435 (15)	0.0153 (13)	0.0166 (12)	0.0060 (13)
C14	0.0346 (14)	0.0347 (14)	0.074 (2)	0.0021 (11)	0.0089 (13)	0.0009 (13)
C15	0.0343 (13)	0.0351 (13)	0.0604 (17)	0.0018 (11)	0.0107 (12)	−0.0027 (12)
C16	0.0293 (14)	0.0547 (19)	0.097 (3)	0.0095 (13)	0.0075 (16)	0.0062 (18)
C17	0.0551 (18)	0.0295 (13)	0.0676 (19)	0.0070 (12)	0.0177 (15)	−0.0009 (13)
C18	0.0523 (18)	0.0429 (16)	0.071 (2)	0.0222 (14)	0.0174 (15)	0.0094 (14)
C19	0.0419 (14)	0.0368 (13)	0.0467 (15)	−0.0030 (11)	0.0157 (12)	−0.0055 (11)
C20	0.0361 (13)	0.0458 (15)	0.0473 (15)	−0.0007 (11)	0.0140 (12)	−0.0021 (12)
C21	0.0442 (16)	0.0507 (16)	0.0422 (15)	0.0023 (13)	0.0082 (12)	−0.0080 (12)
C22	0.0504 (17)	0.0507 (16)	0.0545 (17)	−0.0018 (13)	0.0244 (14)	0.0044 (14)
C23	0.0555 (17)	0.0479 (16)	0.0425 (15)	0.0132 (13)	0.0172 (13)	0.0035 (12)

### Geometric parameters (Å, °)

**Table:**

Br1—C5	1.899 (2)	C8—H8	0.9300
Ni1—O3^i^	1.9963 (16)	C9—C11	1.384 (4)
Ni1—N2	2.1439 (19)	C9—H9	0.9300
Ni1—O2	2.1655 (17)	C10—C12	1.368 (4)
Ni1—O1	2.2024 (17)	C10—H10	0.9300
Ni1—N1	2.204 (2)	C11—C13	1.362 (4)
Ni1—N3	2.204 (2)	C11—H11	0.9300
O1—C1	1.264 (3)	C12—C13	1.377 (4)
N1—C9	1.336 (3)	C12—H12	0.9300
N1—C10	1.345 (3)	C13—H13	0.9300
C1—O2	1.259 (3)	C14—C16	1.380 (4)
C1—C7	1.496 (3)	C14—H14	0.9300
N2—C14	1.318 (3)	C15—C17	1.377 (4)
N2—C15	1.330 (3)	C15—H15	0.9300
C2—O4	1.226 (3)	C16—C18	1.363 (5)
C2—O3	1.282 (3)	C16—H16	0.9300
C2—C3	1.511 (3)	C17—C18	1.373 (4)
O3—Ni1^ii^	1.9962 (16)	C17—H17	0.9300
N3—C20	1.335 (3)	C18—H18	0.9300
N3—C19	1.341 (3)	C19—C21	1.375 (4)
C3—C8	1.381 (3)	C19—H19	0.9300
C3—C4	1.390 (3)	C20—C22	1.386 (4)
C4—C5	1.381 (3)	C20—H20	0.9300
C4—H4	0.9300	C21—C23	1.372 (4)
C5—C6	1.380 (3)	C21—H21	0.9300
C6—C7	1.390 (3)	C22—C23	1.383 (4)
C6—H6	0.9300	C22—H22	0.9300
C7—C8	1.387 (3)	C23—H23	0.9300
			
O3^i^—Ni1—N2	94.29 (7)	C3—C8—C7	121.0 (2)
O3^i^—Ni1—O2	112.73 (7)	C3—C8—H8	119.5
N2—Ni1—O2	152.98 (7)	C7—C8—H8	119.5
O3^i^—Ni1—O1	172.84 (7)	N1—C9—C11	123.7 (3)
N2—Ni1—O1	92.78 (7)	N1—C9—H9	118.2
O2—Ni1—O1	60.20 (6)	C11—C9—H9	118.2
O3^i^—Ni1—N1	88.82 (7)	N1—C10—C12	123.4 (3)
N2—Ni1—N1	90.11 (7)	N1—C10—H10	118.3
O2—Ni1—N1	90.48 (7)	C12—C10—H10	118.3
O1—Ni1—N1	90.02 (7)	C13—C11—C9	119.1 (3)
O3^i^—Ni1—N3	90.39 (7)	C13—C11—H11	120.5
N2—Ni1—N3	91.78 (7)	C9—C11—H11	120.5
O2—Ni1—N3	88.13 (7)	C10—C12—C13	119.4 (3)
O1—Ni1—N3	90.54 (7)	C10—C12—H12	120.3
N1—Ni1—N3	178.00 (7)	C13—C12—H12	120.3
C1—O1—Ni1	88.74 (14)	C11—C13—C12	118.3 (3)
C9—N1—C10	116.1 (2)	C11—C13—H13	120.9
C9—N1—Ni1	124.07 (17)	C12—C13—H13	120.9
C10—N1—Ni1	119.51 (17)	N2—C14—C16	123.8 (3)
O2—C1—O1	120.5 (2)	N2—C14—H14	118.1
O2—C1—C7	119.7 (2)	C16—C14—H14	118.1
O1—C1—C7	119.7 (2)	N2—C15—C17	123.3 (3)
C1—O2—Ni1	90.54 (14)	N2—C15—H15	118.4
C14—N2—C15	117.1 (2)	C17—C15—H15	118.4
C14—N2—Ni1	118.52 (17)	C18—C16—C14	118.4 (3)
C15—N2—Ni1	124.42 (17)	C18—C16—H16	120.8
O4—C2—O3	125.9 (2)	C14—C16—H16	120.8
O4—C2—C3	119.5 (2)	C18—C17—C15	118.5 (3)
O3—C2—C3	114.5 (2)	C18—C17—H17	120.8
C2—O3—Ni1^ii^	130.29 (15)	C15—C17—H17	120.8
C20—N3—C19	116.3 (2)	C16—C18—C17	119.0 (3)
C20—N3—Ni1	122.15 (17)	C16—C18—H18	120.5
C19—N3—Ni1	121.10 (17)	C17—C18—H18	120.5
C8—C3—C4	119.5 (2)	N3—C19—C21	124.0 (3)
C8—C3—C2	121.0 (2)	N3—C19—H19	118.0
C4—C3—C2	119.5 (2)	C21—C19—H19	118.0
C5—C4—C3	119.2 (2)	N3—C20—C22	123.4 (3)
C5—C4—H4	120.4	N3—C20—H20	118.3
C3—C4—H4	120.4	C22—C20—H20	118.3
C6—C5—C4	121.8 (2)	C23—C21—C19	119.1 (3)
C6—C5—Br1	119.11 (17)	C23—C21—H21	120.4
C4—C5—Br1	119.06 (17)	C19—C21—H21	120.4
C5—C6—C7	118.8 (2)	C23—C22—C20	119.1 (3)
C5—C6—H6	120.6	C23—C22—H22	120.5
C7—C6—H6	120.6	C20—C22—H22	120.5
C8—C7—C6	119.7 (2)	C21—C23—C22	118.1 (3)
C8—C7—C1	120.1 (2)	C21—C23—H23	120.9
C6—C7—C1	120.1 (2)	C22—C23—H23	121.0
			
N2—Ni1—O1—C1	179.66 (14)	O4—C2—C3—C4	2.8 (3)
O2—Ni1—O1—C1	0.32 (13)	O3—C2—C3—C4	−175.6 (2)
N1—Ni1—O1—C1	−90.23 (14)	C8—C3—C4—C5	−0.3 (3)
N3—Ni1—O1—C1	87.85 (14)	C2—C3—C4—C5	−179.8 (2)
O3^i^—Ni1—N1—C9	−146.9 (2)	C3—C4—C5—C6	0.1 (4)
N2—Ni1—N1—C9	118.8 (2)	C3—C4—C5—Br1	−179.01 (17)
O2—Ni1—N1—C9	−34.2 (2)	C4—C5—C6—C7	0.0 (4)
O1—Ni1—N1—C9	26.0 (2)	Br1—C5—C6—C7	179.19 (17)
O3^i^—Ni1—N1—C10	26.11 (19)	C5—C6—C7—C8	−0.1 (3)
N2—Ni1—N1—C10	−68.18 (19)	C5—C6—C7—C1	−178.3 (2)
O2—Ni1—N1—C10	138.84 (19)	O2—C1—C7—C8	−0.3 (3)
O1—Ni1—N1—C10	−160.96 (18)	O1—C1—C7—C8	−179.3 (2)
Ni1—O1—C1—O2	−0.5 (2)	O2—C1—C7—C6	177.9 (2)
Ni1—O1—C1—C7	178.45 (19)	O1—C1—C7—C6	−1.1 (3)
O1—C1—O2—Ni1	0.6 (2)	C4—C3—C8—C7	0.2 (3)
C7—C1—O2—Ni1	−178.44 (19)	C2—C3—C8—C7	179.8 (2)
O3^i^—Ni1—O2—C1	178.35 (13)	C6—C7—C8—C3	0.0 (3)
N2—Ni1—O2—C1	−1.8 (2)	C1—C7—C8—C3	178.2 (2)
O1—Ni1—O2—C1	−0.32 (13)	C10—N1—C9—C11	−2.0 (4)
N1—Ni1—O2—C1	89.43 (14)	Ni1—N1—C9—C11	171.2 (2)
N3—Ni1—O2—C1	−92.01 (14)	C9—N1—C10—C12	1.8 (4)
O3^i^—Ni1—N2—C14	−174.3 (2)	Ni1—N1—C10—C12	−171.7 (2)
O2—Ni1—N2—C14	5.8 (3)	N1—C9—C11—C13	0.0 (4)
O1—Ni1—N2—C14	4.6 (2)	N1—C10—C12—C13	0.4 (4)
N1—Ni1—N2—C14	−85.5 (2)	C9—C11—C13—C12	2.3 (4)
N3—Ni1—N2—C14	95.2 (2)	C10—C12—C13—C11	−2.5 (4)
O3^i^—Ni1—N2—C15	6.8 (2)	C15—N2—C14—C16	0.3 (5)
O2—Ni1—N2—C15	−173.11 (19)	Ni1—N2—C14—C16	−178.7 (3)
O1—Ni1—N2—C15	−174.4 (2)	C14—N2—C15—C17	0.9 (4)
N1—Ni1—N2—C15	95.6 (2)	Ni1—N2—C15—C17	179.8 (2)
N3—Ni1—N2—C15	−83.7 (2)	N2—C14—C16—C18	−1.0 (6)
O4—C2—O3—Ni1^ii^	−0.5 (4)	N2—C15—C17—C18	−1.3 (5)
C3—C2—O3—Ni1^ii^	177.90 (14)	C14—C16—C18—C17	0.5 (5)
O3^i^—Ni1—N3—C20	175.21 (19)	C15—C17—C18—C16	0.6 (5)
N2—Ni1—N3—C20	−90.49 (19)	C20—N3—C19—C21	−1.2 (4)
O2—Ni1—N3—C20	62.47 (19)	Ni1—N3—C19—C21	171.5 (2)
O1—Ni1—N3—C20	2.31 (19)	C19—N3—C20—C22	1.4 (4)
O3^i^—Ni1—N3—C19	2.84 (19)	Ni1—N3—C20—C22	−171.3 (2)
N2—Ni1—N3—C19	97.14 (19)	N3—C19—C21—C23	−0.1 (4)
O2—Ni1—N3—C19	−109.90 (19)	N3—C20—C22—C23	−0.1 (4)
O1—Ni1—N3—C19	−170.06 (18)	C19—C21—C23—C22	1.4 (4)
O4—C2—C3—C8	−176.7 (2)	C20—C22—C23—C21	−1.3 (4)
O3—C2—C3—C8	4.8 (3)		

Symmetry codes: (i) −*x*+3/2, *y*−1/2, −*z*+3/2; (ii) −*x*+3/2, *y*+1/2, −*z*+3/2.
